# Crystal structure of a DNA polymerase sliding clamp from a Gram-positive bacterium

**DOI:** 10.1186/1472-6807-6-2

**Published:** 2006-01-10

**Authors:** Maria A Argiriadi, Eric R Goedken, Irina Bruck, Mike O'Donnell, John Kuriyan

**Affiliations:** 1 Howard Hughes Medical Institute, The Rockefeller University, 1230 York Avenue, New York, NY 10021, USA; 2 Howard Hughes Medical Institute, Dept. of Molecular and Cell Biology, Dept. of Chemistry, University of California, Berkeley, CA 94720, USA; 3 Physical Biosciences Division, Lawrence Berkeley National Laboratory, Berkeley, CA 94720, USA

## Abstract

**Background:**

Sliding DNA clamps are processivity factors that are required for efficient DNA replication. DNA polymerases maintain proximity to nucleic acid templates by interacting with sliding clamps that encircle DNA and thereby link the polymerase enzyme to the DNA substrate. Although the structures of sliding clamps from Gram-negative bacteria (*E. coli*), eukaryotes, archaea, and T4-like bacteriophages are well-known, the structure of a sliding clamp from Gram-positive bacteria has not been reported previously.

**Results:**

We have determined the crystal structure of the dimeric β subunit of the DNA polymerase III holoenzyme of *Streptococcus pyogenes*. The sliding clamp from this Gram-positive organism forms a ring-shaped dimeric assembly that is similar in overall structure to that of the sliding clamps from Gram-negative bacteria, bacteriophage T4, eukaryotes and archaea. The dimer has overall dimensions of ~90 Å × ~70 Å × ~25 Å with a central chamber that is large enough to accommodate duplex DNA. In comparison to the circular shape of other assemblies, the *S. pyogenes *clamp adopts a more elliptical structure.

**Conclusion:**

The sequences of sliding clamps from *S. pyogenes *and *E. coli *are only 23% identical, making the generation of structural models for the *S. pyogenes *clamp difficult in the absence of direct experimental information. Our structure of the *S. pyogenes *β subunit completes the catalog of clamp structures from all the major sequence grouping of sliding clamps. The more elliptical rather than circular structure of the *S. pyogenes *clamp implies that the topological nature of encircling DNA, rather than a precise geometric shape, is the most conserved aspect for this family of proteins.

## Background

The bacterial DNA Polymerase III holoenzyme has the remarkable ability to polymerize long stretches of DNA at great speeds (~750 bases per second for the *E. coli *enzyme) without dissociating from its template [1-3]. The β subunit of the holoenzyme is required for efficient processivity in bacteria. This protein factor wraps around double-stranded DNA at primer-template junctions, where it serves as a sliding clamp to tether the polymerase enzyme to its DNA substrate (reviewed in [4-6]). A separate ATP-dependent protein complex called the clamp loader is required to place the sliding clamp on primer-template DNA (reviewed in [7-9]). Once loaded by this complex, the sliding clamp can confer processivity to the catalytic α subunit of the polymerase.

Structural analysis of the *E. coli *β subunit showed that the bacterial clamp is a head-to-tail dimer, with two protomers that form a closed ring [10]. The diameter of the ring interior is large enough to accommodate duplex DNA. Biochemical studies indicate that one interface of the clamp is opened by the clamp loader to allow passage of a primed DNA into this interior [11]. The bacterial clamp does not share readily detectable sequence similarity with the trimeric proliferating cellular nuclear antigen (PCNA) clamps of eukaryotes and archaea. Nevertheless, the overall ring-shaped architecture of sliding clamps is highly similar in bacterial, archaeal, and eukaryotic clamps [12-14] as well as those from bacteriophage [15,16].

**Figure 1:**
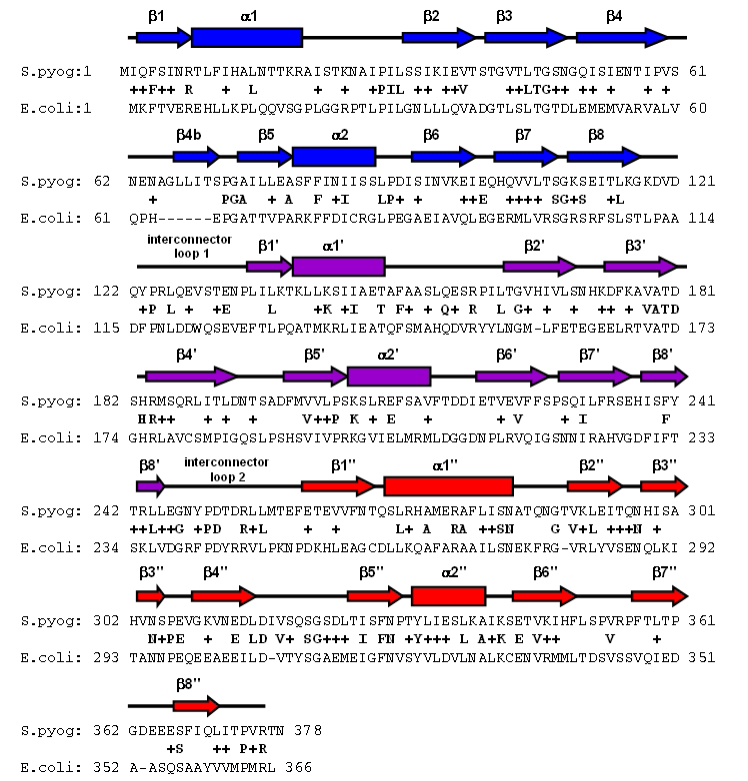
Alignment of the sequences of the *Streptococcus pyogenes *and *E. coli *β subunits as done by BLAST . Secondary structure elements are labeled using the nomenclature first used for *E. coli *β in ref. [10]. An additional β-strand found only in domain I of the *S. pyogenes *clamp from residues 67–71 is labeled as β4b.

**Figure 2:**
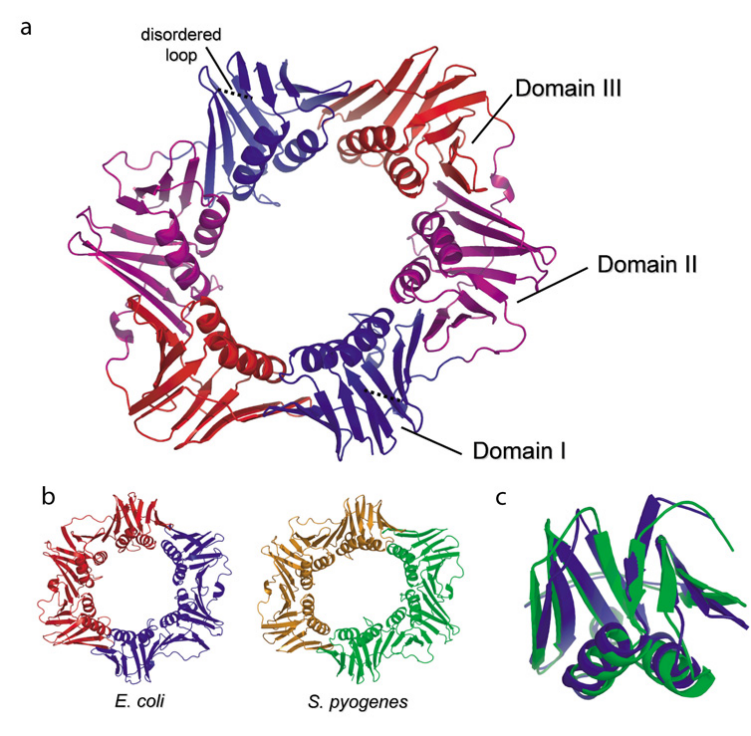
(A) Structure of the *Streptococcus pyogenes *β subunit. Ribbon representation of the *S. pyogenes *β subunit. (B) Comparison of *S. pyogenes *β subunit with *E. coli *β [10]. (C) Overlay of domain II of *S. pyogenes *(green) and *E. coli *(blue) β. Superposition was done with top3d [25], and figures were rendered with PyMol (DeLano Scientific).

Here we present the crystal structure of the β subunit of DNA polymerase III from *Streptococcus pyogenes*, a Gram-positive bacterium. The subunits of the replicative machinery of Gram-positive bacteria are generally divergent in sequence from those of Gram-negative counterparts such as *E. coli *[17]. The β subunit of *S. pyogenes *shares only 23% sequence identity to the *E. coli *protein (Figure 1). We find that while the *S. pyogenes *clamp has a fold that is strikingly similar to that of the sliding clamp from *E. coli*, the *S. pyogenes *clamp adopts a more elliptical shape (Figure 2). This indicates that although the *S. pyogenes *clamp presumably shares ability to encircle DNA, the precise details of the molecular shape of sliding clamps are poorly conserved between Gram-positive and Gram-negative bacteria.

## Results and discussion

The crystal structure of the β subunit from *Streptococcus pyogenes *was determined by multiple anomalous diffraction (MAD) at 2.6 Å resolution using datasets for three separate wavelengths close to the selenium absorption edge. A model built into the phased electron density maps was refined subsequently to 2.1 Å resolution against native data (See Materials & Methods). These crystals (space group P2_1_) contain one β dimer in the asymmetric unit. A non-crystallographic two-fold relates two protomers to form the dimeric clamp. This internal symmetry facilitated the initial stages of model building and refinement. The two molecules are related by a 180° rotation about an axis perpendicular to the plane of the ring with a root mean square (r.m.s.) deviation of 0.46 Å for Cα atoms between the two protomers.

Consistent with the structure of the *E. coli *β clamp [10], the *S. pyogenes *clamp dimer is formed in a head-to-tail fashion. The sequence identity between the *E. coli *and *S. pyogenes *β clamp sequences is relatively low at 23% (Figure 1). Low sequence identity is a characteristic of sequence comparisons between bacterial clamps and trimeric clamps, such as those of T4-bacteriophage gp45 and PCNA [12-16]. Interestingly, the *S. pyogenes *β clamp dimer has a distorted oval shape, unlike the more circular shape of the *E. coli *β clamp. The distortion from a more circular shape is a feature of both crystallographically-independent subunits although we cannot absolutely rule out the influence of crystal packing on the shape of these clamps. The longer of the outer oval diameters is ~90 Å while the shortest diameter across the clamp is ~65 Å (Figure 2).

The overall organization of the *S. pyogenes *β subunit is very similar to that of the *E. coli *β subunit, with three distinct domains in each protomer. The secondary structure and topology of each of these domains is also very similar to each other; a pair of four-stranded antiparallel β sheets that bracket two antiparallel α helices make up the fundamental domain that is repeated three times per protomer (and six times per clamp). Domains II and III consist of approximately 110 residues. Domain I contains six more residues that are inserted in a loop region between strands β4 and β5. This region includes a small additional β-strand (β4b) not present in the *E. coli *structure (residues 66–70) and several residues that are disordered (residues 61–65) in both protomers of the *S. pyogenes *clamp. The additional β-strand is part of an antiparallel five-stranded β-sheet made up together of strands β1, β4b, β6, β7 and β8. We note that a region comparable to residues 66–70 of the *S. pyogenes *clamp forms a fifth β-strand in the structure of yeast PCNA [12].

When comparing the *E. coli *and *S. pyogenes *β clamp structures, there are differences in the relative positioning of domains and their respective interconnecting linkers that contribute to the elliptical shape of the *S. pyogenes *clamp. This results in an overall r.m.s. deviation for Cα atoms that is relatively large (2.0 Å) between the β clamp dimers from *E. coli *and *S. pyogenes*. The r.m.s.d. in Cα positions of the individual domains that make up the basic clamp fold is smaller (~1.4 Å; an overlay of domains II of the *E. coli *and *S. pyogenes *clamps is shown in Figure 2c).

**Figure 3:**
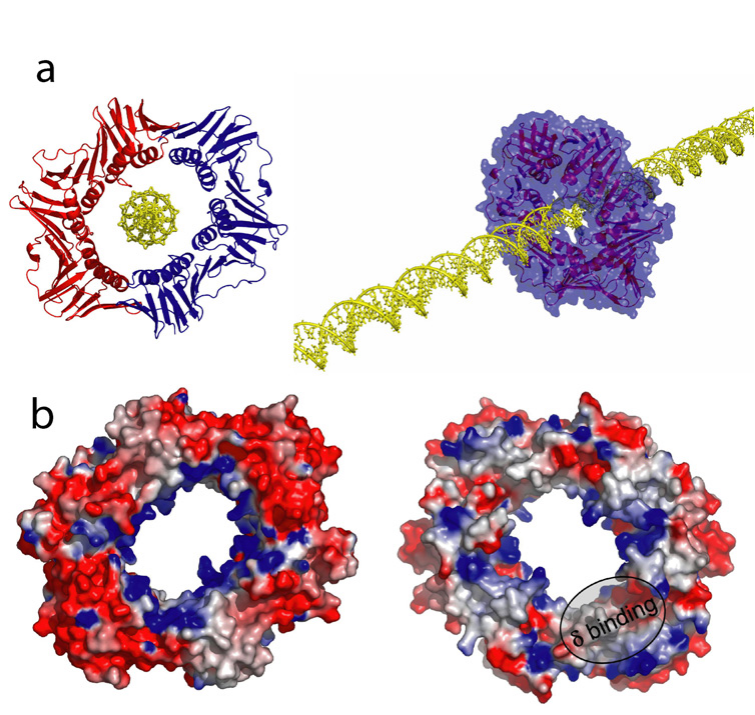
(A) Model of *Streptococcus pyogenes *β clamp surrounding DNA. (B) Electrostatic surface of *S. pyogenes *β subunit. Two views of the clamp are shown that differ by a ~180° rotation about the vertical axis. Red indicates regions of negative electrostatic potential, white indicates neutral regions, and blue indicates positive regions. Figure rendered with GRASP [26, 27] and PyMol (DeLano Scientific). The position on the face of the *S. pyogenes *clamp where the δ subunit of the clamp loader would be expected to bind is indicated based upon the co-crystal structure of the *E. coli *proteins [18].

Despite the differences in structure, the *S. pyogenes *clamp, like that of *E. coli*, can readily accommodate duplex DNA within it (Figure 3a). The width of the central chamber is ~41 Å at its longest dimension and ~30 Å at its shortest dimension. As for other clamps, the electrostatic properties of the *S. pyogenes *clamp are a striking aspect of the structure (Figure 3b). The interior of the central hole in the *S. pyogenes *clamp is lined with basic residues, many of which (e.g., Arg 8, Lys 25, Lys 143, Lys 206, Arg 272, and Lys 342) are conserved between the *S. pyogenes *and the *E. coli *proteins. While these residues provide a positively-charged region of the clamp for interaction with DNA, the remainder of the protein is largely acidic; the protein has a calculated isoelectric point (pI) of 5.4. Most of the negatively-charged patches on the surface of the *S. pyogenes *β subunit are found on the face of the clamp that is opposite to the predicted site of interaction with the clamp loader and polymerase (Figure 3b).

**Figure 4:**
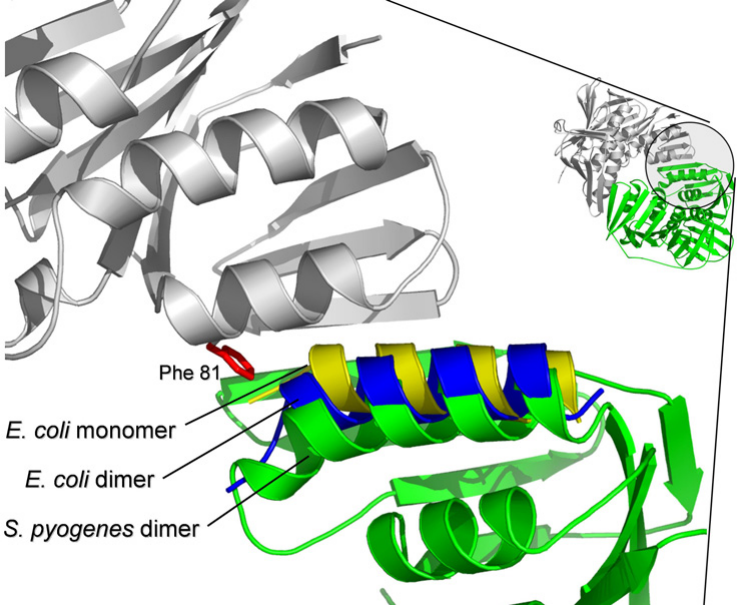
Differences in helix α1" geometry between in *E. coli *and *S. pyogenes *β. The dimeric interface of both β clamps distort of this helix (*E. coli *in blue, *S. pyogenes in *green). In the monomeric form of β (shown in yellow) crystallized bound to the δ subunit of the clamp loader [18], this helix is straight. The other protomer of *S. pyogenes *β is shown in grey for reference, with the sidechain of Phe81 shown in red. Figure rendered with with PyMol (DeLano Scientific).

There is an interesting feature that is common to the dimeric interfaces of the structures of the *S. pyogenes *and *E. coli *clamps. In the *E. coli *clamp structure, helix α1" (see Figure 1 for notation) exhibits distorted helical geometry in order to maintain the integrity of the dimeric interface [18] (Figure 4). The distorted helix is kinked over a region spanning three residues at its C-terminus (Ala 271, Ile 272, Leu 273). This pronounced distortion appears to be correlated with the formation of an intermolecular ion pair (between Lys 74 and Glu 300) at this interface. In contrast, the α1" helix is straightened out in a monomeric form of the *E. coli *β clamp complexed with a clamp loader subunit (δ) responsible for opening the clamp [18]. This observation led to the idea that the changes at the clamp interface in helix α1" may be part of a "spring-loaded" mechanism for clamp opening [18]. In this hypothesis, the energetic strain of distorting the helical geometry at the dimeric interface is released upon interaction with the δ subunit of the clamp loader complex. In the closed form of the *S. pyogenes *β clamp, helix α1" displays an even larger displacement from ideal geometry in comparison to the *E. coli *β clamp (Figure 4). This distortion of the interface in the *S. pyogenes *clamp is correlated with the presence of the sidechain of Phe 81, which pushes against helix α1" (Figure 4). Thus, although the molecular determinants of this helical distortion are different in the two proteins, it appears that the structural effect is conserved. Interestingly, the *S. pyogenes *DNA polymerase III can utilize the *E. coli *β clamp to achieve processivity, despite the low sequence identity between the clamps and the difference in overall shape. However, the *E. coli *polymerase III is unable make reciprocal use of the *S. pyogenes *clamp [17].

## Conclusion

The oval shape of the *S. pyogenes *clamp suggests that it is the topological property of surrounding the DNA template in order to maintain DNA-interactions with its cognate polymerase that is a fundamental and conserved aspect of bacterial β clamps. The subunit organization and secondary structural topology of the *S. pyogenes *clamp is very similar to that of the *E. coli *sliding clamp. Like in the *E. coli *clamp, the *S. pyogenes *subunits form a head-to tail dimmer, with a markedly positive electrostatic potential within the interior of the ring. The *S. pyogenes *clamp is therefore expected to readily accommodate duplex DNA within it.

## Methods

### Sample preparation and characterization

The *Streptococcus pyogenes *β subunit was overexpressed in BLR DE3 cells using a previously described expression plasmid (pETSpdnaN, [17]). Wild-type β subunit was grown in 12 liters LB broth at 37°C and selenomethionine-derivatized β was grown in 8 liters of minimal media supplemented with selenomethionine at 37°C. Both wild-type and selenomethionine cells were selected with the inclusion of ampicillin and induced with 1 mM IPTG at 15°C for 12–14 hours. The cells were resuspended, and lysed via French press in Buffer A (20 mM Tris pH 7.5, 0.1 mM EDTA, 10% glycerol, 5 mM DTT) and 1 M NaCl. In all subsequent purification steps, fresh 5 mM DTT was added to prevent oxidative degradation. The lysate was initially purified by an ammonium sulfate cut (0.3 grams of ammonium sulfate per mL of lysate). The pellet was then resuspended in Buffer A + 20 mM NaCl and dialyzed overnight against 2 L of the same buffer mixture. Protein was eluted through a Fast Flow Q column over a 10-column volume gradient from Buffer A + 50 mM NaCl to Buffer A + 500 mM NaCl. The pooled protein was diluted two-fold and eluted through an EAH-Sepharose 4B column over a 10-column volume gradient from Buffer A + 50 mM NaCl to Buffer A + 500 mM NaCl. *S. pyogenes *β fractions were pooled and precipitated again with ammonium sulfate to minimize the volume of eluted sample. The pellet was resuspended and dialyzed to adjust conductivity. An additional ion exchange step (Mono Q) removed minor impurities using a 10-column volume gradient from Buffer A + 50 mM NaCl to Buffer A + 500 mM NaCl. Size exclusion (Superdex 200) was a final purification step used only for the selenomethionine-derivatized protein. Wild-type and selenomethionine purified β subunit was concentrated to 20 mg/mL as determined by Bradford assay.

### Crystallization and x-ray crystallography

Recombinant wild-type and selenomethionine *Streptococcus *β subunit were crystallized by equilibrating 1.0 μL of protein solution (20 mg/mL *Streptococcus *β subunit, Buffer A + 100 mM NaCl) with 1.0 μL of precipitant buffer (30% PEG 2000 MME, 100 mM sodium acetate pH 4.5, 200 mM ammonium sulfate) in a hanging drop suspended over a 1 mL reservoir of precipitant buffer at 20°C. Trapezoidal crystals with approximate dimensions 0.5 mm × 0.2 mm × 0.1 mm appeared within 5 days and diffracted X-rays to 2.1 Å resolution with synchrotron radiation. *S. pyogenes *β subunit crystallized in the spacegroup P2_1 _having unit cell dimensions a = 79.1 Å, b = 74.7 Å, c = 82.8 Å, α = 90°, β = 118.6°, γ = 90° with one dimer in the asymmetric unit and 53.8% estimated solvent content.

Diffraction data were collected from 20% ethylene glycol flashed-cooled crystals by using synchrotron radiation at the Lawrence Berkeley National Laboratories, beamline 5.0.1. Data reduction was achieved using HKL2000 [19]. Attempts to determine the structure by using the crystal structure of *E. coli *β subunit [10] by molecular replacement were unsuccessful due to low sequence identity. Experimental phases were determined using multiple anomalous diffraction (MAD) data collected at 2.5 Å resolution at three wavelengths close to the selenium absorption edge. Selenium atom positions were identified using the program Shake-and-Bake [20] and further refined with the program MLPHARE in the CCP4 suite [21] (8 selenium sites in total, 4 from each monomer). By using the structure of the *E. coli *β subunit [10] and the selenium-derived experimental phases, a phased translation function was calculated using CNS [22] to initially place a model into experimental density, calculated with solvent-flattened non-crystallographic symmetry averaged MAD phases at 3.0 Å resolution using the program DM [23]. Domains I, II, and III of *Streptococcus *β were refined initially as rigid bodies at 3.0 Å. Subsequently, the model was rebuilt and refined against native 2.1 Å data using CNS [22] and O [24]. Individual B-factors were refined and bulk solvent correction was applied, and 145 water molecules were built into the model. Because of disorder in the side-chains for residues Arg 254 and Thr 377 in one protomer, only main-chain atoms were included in the model. The final free and working R-values are 28.4% and 24.5% respectively. Coordinates have been deposited with the Protein Data Bank under the accession code 2AVT.

## Contribution of authors

MAA carried out the expression, purification, crystallization, X-ray data collection and of the refinement of the *S. pyogenes *β clamp, and wrote the initial version of the manuscript. ERG finalized refinement of the structure, further developed the manuscript, and created the manuscript figures. IB cloned the protein expression construct and assisted with protein purification. MOD and JK conceived of the study, participated in its design and coordination and helped to draft the manuscript. All authors read and approved the final manuscript.
